# White adipose tissues and skeletal muscles as a target of chrysin during the treatment of obesity in rats

**DOI:** 10.1038/s41598-024-82820-x

**Published:** 2025-01-06

**Authors:** Omar S. Hassan, Magda A. Megahed, Nesma A. Ghazal

**Affiliations:** Department of Biochemistry, Medical Research Institute, Alexandria University, 165 El-Horreya Avenue, EL-Hadara, POB 21561, Alexandria, Egypt

**Keywords:** White adipose tissue, Skeletal muscle, PPAR-γ, Mir-27a, TNF α, Obesity, Rats, Chrysin, Biochemistry, Biotechnology, Molecular biology

## Abstract

Obesity is a rapidly growing epidemic that continues to be a major severe health problem due to its association with various adverse health consequences. Since 1975, the WHO estimates that the prevalence of obesity has tripled globally. Chrysin is a flavone that is mostly found in the Passiflore species of plants and in propolis. The present study was conducted to examine the anti-obesity effect of chrysin on a high-fat diet-induced obesity in rats and to compare their impact to physical activity (swimming). Rats were classified into two groups: the control group and the obese group, which was subdivided into 4 subgroups (8 rats each); an untreated obese group; a chrysin-treated group (oral dose of 100 mg/kg/day); swimming-treated groups (swimming for 1 h/day, five days/week with a constant overload equal to 5% of their weight); and combination-treated groups (chrysin and swimming). After 8 weeks of treatment, blood samples were taken at the end of the experiment for biochemical tests. Animals were then slaughtered to get white adipose tissue (WAT) and skeletal muscle for analysis of the gene expression of the studied genes. In obese rats, therapy with chrysin reduced weight gain, hyperglycaemia, and insulin resistance. Also, the effects of chrysin may be mediated through acting as an anti-inflammatory and antioxidative stress agent. Physical activity (swimming) is a more efficient anti-obesity agent than treating with chrysin alone through upregulation of PPAR-γ and downregulation of Mir-27a. Physical activity with daily supplementation of chrysin showed the best efficiency for the treatment of obesity.

## Introduction

Obesity is a complex chronic disease that has emerged as a major global public health issue due to its high prevalence, causal relationship with numerous serious medical conditions, negative effects on quality of life, and significant economic consequences related to increased health care costs and decreased productivity. Furthermore, obesity has drawn a lot of attention in the health sciences, and numerous research studies have concentrated not only on the prevalence and risk factors of obesity but also on the important effects on patients’ quality of life^1^.

The World Health Organization (WHO) categorises obesity as a chronic, serious condition that affects both adults and children and is present in both industrialised and developing nations. Although specialised public health strategies and treatment initiatives have been created to combat the obesity epidemic, the prevalence of obesity has increased at a concerning pace^2^, potentially increasing the proportion of people who suffer from obesity-related problems, affecting almost a third of the world’s population now, combined with being overweight. According to estimates, 38% of people worldwide will be overweight and another 20% will be obese by 2030 if current trends continue. About 25% of Egyptians reported having a normal weight, according to a survey conducted as part of the “100 million Health” project, while the remaining 75% were obese or overweight^3^.

Scientists have discovered natural ways to reduce the risks associated with obesity instead of using drugs and artificial methods; much attention has been given to chrysin (5,7-di-OH-flavone), a flavone found in propolis and many plants, primarily in the Passiflora genus. It has many pharmaceutical benefits, including anti-inflammatory, neuroprotective, antidiabetic, anti-atherogenic, hepatoprotective, antitumor, nephroprotective, and cardioprotective properties^4^. According to some research, chrysin may be investigated as a potentially effective dietary addition for preventing obesity because it induces the brown-like phenotype and improves lipid metabolism, also chrysin is highlighted for its potential therapeutic benefits in managing obesity by influencing fat accumulation and improving muscle health^5^.

Traditional medicinal herbs and their constituents, including curcumin, ginsenoside, celastrol, berberine, artemisinin, and capsaicin, have been tested and practiced over the years. These are natural Chinese herb-based treatments for obesity. Curcumin, a key active element in traditional Chinese turmeric (Curcuma longa), has a variety of pharmacological actions, including antioxidant, anti-inflammatory, antiviral, antibacterial, and anticancer properties^6^.

Exercise training has been demonstrated to reduce chronic, low-grade systemic inflammation in human and animals and is regarded as a significant environmental component involved with body weight management. Therefore, raising physical exercise has emerged as a key component of a non-pharmacological approach to managing weight gain and obesity^7^.

Non-coding RNA molecules called microRNA (miRNA) have a length of 22 to 25 nucleotides and are able to control the activation of target genes after transcription, which eventually results in the degradation of target mRNA. MiRNAs are essential for many molecular and biological processes, including cell division, migration, necrocytosis, and death, according to mounting evidence^8,9^. MiR-27a is regarded as one of the most important miRNAs found so far due to its role in the regulation of a variety of biochemical and pathologic processes, including the prevention of osteoarthritis, pancreas cancer, stomach cancer, and human hepatocellular cancer^10^.

As an anti-inflammatory factor, peroxisome proliferator-activated receptor gamma (PPAR-γ) can be used to speed up fatty acid breakdown and lower blood cholesterol levels. By improving peripheral insulin sensitivity and lowering liver glucose synthesis, PPAR-γ activation lowers hyperglycemia. For insulin sensitivity, MiR-27a has been shown to engage in the signaling networks important for glucose metabolism in insulin resistance. Additionally, it has been noted that exosomal miR-27a from adipocytes causes insulin resistance in skeletal muscle by suppressing PPAR-γ expression^11^.

This study aimed to explore the anti-obesity effect of chrysin in a rat model of obesity with a specific dose for two months. In addition, the study aimed to compare the effects of chrysin and/or exercise training through molecular determination of gene expression of Mir27a and PPAR-γ in WAT and skeletal muscle.

## Materials and methods

### Constituents of diets

The control and obesogenic diets are presented in Table 1 as follow:

**Table 1:** Composition of the diets used in the study.

Macronutrients (g/kg diet)	Control diet^12^	Obesogenic diet^12^
Protein		
Casein	220	144
Carbohydrates		
Corn starch	631	150
Dextrose		163
Sucrose		280
Fat		
Lard	–	148
Corn oil	43	20
Cellulose	54	50
Vitamin mix	10	10
Mineral mix	40	35
Total energy (kcal/g diet)	3.8	4.5

### Experimental animals

The study was conducted on 40 young male albino rats 2 months old (100–120 g) obtained from the animal house of Misr University for Science & Technology, Egypt. All rats have free access to food and water with 12:12 h light/dark cycle and constant environmental conditions before experimentation.

### Ethical statement

All experiments pursued the standards of the National Institutes of Health Guide for the Care and Use of Laboratory Animals (NIH Publications No. 8023, revised 1978). The experimental protocol was approved by The Institutional Animal Care and Use Committee (IACUC)-Alexandria University, Egypt. The approval number is AU0122021111. The study also follows ARRIVE guidelines and complies with the National Research Council’s guide for the care and use of laboratory animals.

### Obesity induction

Obesity was induced in rats by feeding them with an obesogenic diet for 3 months as presented in Table 1. Rats that were be 20% heavier than control rats of the same age were considered obese^12^.

### Treatment of obesity

Chrysin was purchased from Sigma Aldrich and given to rats orally by gastric tube in a dose of 100 mg/kg/day for two month^13^.

### Experimental design

**Fig. 1:**
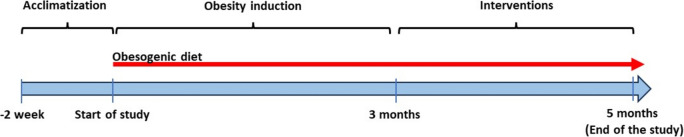
The time-line of the study.

Animals were classified into the following groups: (1) Healthy control group that consists of 8 healthy male rats, after the establishment of obesity, the 32 obese male rats were divided into 4 groups (8 rats each) according to the treatment: (2) Untreated-obese group, (3) Chrysin-treated obese group, that received chrysin orally in a dose of 100 mg/kg daily for two months^13^, (4) Swimming-treated obese group, in which obese rats were trained by swimming for 60 min/day, five days a week for two months, with a constant overload equivalent to 5% of their body weight^14^. (5) Combined treatment-obese group, in which obese rats received chrysin orally in a dose of 100 mg/kg daily and trained by swimming for 60 min/day, five days a week for two months. All treatments were continued for 8 weeks, and all obese rats were maintained under the obesogenic diet during the experimental period. The time-line of the study is indicated in Fig. 1.

### Collection of samples

At the end of the treatment period, the rats were overnight fasted, Blood samples were collected then all rats were sacrificed by deep anesthesia using isoflurane. WAT (Visceral white adipose tissue) and skeletal muscles tissues (from the thigh) were dissected out and were quickly removed, washed with saline, and stored at -80ºC till analyzed. Blood samples were collected from the retro-orbital vein in anticoagulant free tubes^15^. The samples were left for 20 min at room temperature centrifuged at 3000 × g for 10 min to obtain serum for assessment of fasting blood glucose level (FBG), insulin level, lipid profile (triglycerides level (TG), total cholesterol level (TC), high-density lipoprotein-cholesterol (HDL-C) level, Low-density lipoprotein-cholesterol (LDL-C) level, alanine aminotransferase (ALT) activity, aspartate aminotransferase (AST) activity, urea level, creatinine level, tumor necrosis factor α (TNFα) level and Malondialdehyde level (MDA). The tissues of white adipose tissue and skeletal muscle were used for the extraction of total RNA for Quantitative Real Time-Polymerase Chain Reaction (qRT-PCR) analysis for assessment gene expression of peroxisome proliferator-activated receptor-gamma (PPAR-γ) and MiR-27a.

### Serum parameters measurements

Serum insulin concentration was determined following the instructions of the Insulin rat ELISA kit (EMD Millipore, USA), absorbance was measured at 450 nm and the homeostasis model assessment index for insulin resistance (HOMA-IR) was then calculated using the following formula^16^:

$${\text{HOMA - IR}} = \frac{{{\text{Fasting}}\;{\text{insulin}}\left( {\left( {\upmu {\text{IU}}} \right)/{\text{mL}}} \right) \times {\text{Fasting}}\;{\text{glucose}}\left( {{\text{mg}}/{\text{dL}}} \right)}}{22.5 \times 18}$$

Serum TG, TC and HDL–C levels were determined by the enzymatic colorimetric method using reagents obtained from BioMed Diagnostics INC (USA), absorbance was measured at 546 nm. Serum LDL-C was calculated from TG, TC and HDL-C concentrations using the following equation^17^:

$${\text{LDL}} - {\text{C}}\left( {{\text{mg}}/{\text{dL}}} \right) = {\text{TC}}{-}\left( {{\text{HDL}} - {\text{C}}} \right){-}{\text{TG}}/5$$

Serum ALT and AST activities were determined using reagents obtained from BioMed Diagnostics INC (USA), absorbance was measured at 340 nm^16^. Urea and Creatinine were determined using reagents obtained from BioMed Diagnostics INC (USA), absorbance was measured at 570 nm and 510 nm, respectively^18,19^.

Immunoassay kit (EMD Millipore USA) was used for the non-radioactive quantitative quantification of TNF-α level in rat serum^20^.

Malondialdehyde was determined according to the method of Draper and Hadley^21^.

### Gene expression detection of PPAR-γ and Mir 27a

Total RNA was isolated from WAT using RNeasy Mini Kit (Qiagen®, Germany) according to the manufacturer’s instructions and the concentration and integrity of extracted RNA were checked using nanodrop. Reverse transcription was done using Viva cDNA Synthesis Kit (Qiagen) according to the manufacturer’s instructions. The tissue expressions of PPAR-γ and Mir 27a were quantified in the cDNA using Rotor Gene SYBR Green PCR Kit (Qiagen®, USA). Quantitative PCR amplification conditions were adjusted as an initial denaturation at 95 °C for 10 min and then 45 cycles of PCR for amplification as follows: denaturation at 95 °C for 20 s, annealing at 55 °C for 20 s and extension at 70 °C for 15 s. The housekeeping genes β-actin and U6 were used as reference genes for normalization. The primers used for the determination of rat genes are presented in Table 2. The relative change in mRNA expression in samples was estimated using the 2^−ΔΔCt^ method^22^.

**Table 2:** Primers sequence for real time-PCR.

Gene	Primer sequence	
β-actin (Actb) reference gene	F:	5′-ATCATTGCTCCTCCTGAGCG-3′
	R:	5′-GAAAGGGTGTAAAACGCAGCTC-3′
PPAR-γ	F:	5′-GCCGCCTCAGATTTGAAAGAA-3′
	R:	5′-ACAGAGCTGATTCCGAAGTT-3′
Mir 27a	F:	Purchased from qiagen Cat. No. (Qiagen) MS00003241
	R:	miScript universal primer included in the kit (Qiagen)
U6 reference gene for MiRNA	F:	purchased from qiagen Cat. No. (Qiagen):MS00033740
	R:	miScript universal primer included in the kit (Qiagen)

### Statistical analysis

Data were analyzed using SPSS software package version 18.0 (SPSS Chicago, IL, USA). The data were expressed as mean ± standard deviation (SD) and analyzed using one-way analysis of variance (ANOVA) to compare between different groups. The *P*-value was assumed to be significant at *P *< 0.05. The correlation coefficients (r) between different assayed parameters were evaluated using the Pearson correlation coefficient; *P *< 0.05 was considered as the significance limit for all comparisons^23^.

## Results

### Weight change

Before the start of treatments, all the obese rats were significantly heavier than the control rats. After the treatments, all the obese groups were still significantly heavier than the healthy control group; however, their body weight was significantly lower than the untreated obese rats. The untreated obese rats had significantly higher weight gain compared with the healthy control rats, while the other treated obese rats had significantly lower weight gain compared with untreated obese rats (*P *< 0.05). The obese rats treated with a combination of chrysin and swimming showed the best lowering effect on weight gain, as shown in Table 3.

**Table 3:** The changes of initial and final weights and weight gain in the different studied groups:

Groups	Initial weight (g)	Final weight (g)	Weight gain (g)
Control	225.88 ± 7.43	245.63 ± 16.94	19.75 ± 14.02
Untreated	315.25^a^ ± 8.0	385.88^a^ ± 18.42	70.63^a^ ± 13.08
Chrysin treated	311.50^a^ ± 8.19	336.63^ab^ ± 19.18	25.13^b^ ± 12.39
Swimming treated	318.75^a^ ± 6.80	335.0^ab^ ± 15.60	16.25^b^ ± 15.23
Chrysin & swimming treated	309.75^a^ ± 9.39	325.13^ab^ ± 20.17	15.38^b^ ± 20.16

Data were expressed as Mean ± SD, n = 8 *P* < 0.05 considered significant.

Comparison between different studied groups is carried out using Post Hoc Test (Tukey) for ANOVA test.

^a^Significantly different from Control rats.

^b^Significantly different from Untreated obese rats.

### Glucose homeostasis parameters

Untreated obese rats had a significant elevation in glucose homeostasis parameters (FBG, insulin, and HOMA-IR) compared with the healthy control group. The chrysin treatment did not significantly affect FBS, but it significantly affected insulin level and HOMA-IR, which showed a significant reduction compared with untreated obese rats. The treatment of obese rats with swimming alone or in combination with chrysin significantly reduced these parameters compared with the untreated rats (*P *< 0.05). The better effects were observed in the obese rats treated with chrysin combined with swimming, as shown in Table 4.

**Table 4:** The changes of glucose homeostasis parameters in the different studied groups.

Groups	FBS (mg/dl)	Insulin (µIU/ml)	HOMA-IR
Control	112.9 ± 3.64	8.95 ± 0.45	2.50 ± 0.17
Untreated	135.1^a^ ± 11.48	14.95^a^ ± 2.10	4.56^a^ ± 1.43
Chrysin	122.8 ± 11.40	12.71^ab^ ± 0.86	3.86^a^ ± 0.51
Swimming	119.9^b^ ± 6.10	10.21^bc^ ± 0.78	3.02^b^ ± 0.29
Chrysin & swimming	113.5^b^ ± 7.87	9.78^bc^ ± 0.53	2.74^bc^ ± 0.20

FBG: Fasting Blood sugar (mg/dl).

HOMA-IR: Homeostatic Model Assessment for Insulin Resistance.

Data were expressed as Mean ± SD, n = 8 *P* < 0.05 considered significant.

Comparison between different studied groups is carried out using Post Hoc Test (Tukey) for ANOVA test.

^a^Significantly different from Control rats.

^b^Significantly different from Untreated obese rats.

^c^Significantly different from Chrysin treated rats.

### Lipid profile parameters

The levels of TG, total, and LDL-cholesterol were significantly higher, while HDL-cholesterol was significantly lower in the untreated obese rats compared with the healthy control group. The obese rats treated with chrysin showed significantly lower TG, total, and LDL-cholesterol and significantly higher HDL-cholesterol levels compared with the untreated group. Also, the obese rats treated with swimming showed significant improvement on lipid profile to a higher extent than chrysin. The rats treated with a combination of swimming and chrysin showed the best improvements on lipid profile, as it showed a significant reduction in TG, total and LDL-cholesterol and a significant increase in HDL-cholesterol levels compared with untreated groups (*P* < 0.05), as shown in Table 5.

**Table 5:** The changes of lipid profile parameters (HDL-C, LDL-C) in the different studied groups.

Groups	Triglycerides (mg/dl)	Total cholesterol (mg/dl)	HDL-C (mg/dl)	LDL-C (mg/dl)
Control	108.8 ± 3.69	67.50 ± 1.69	35.63 ± 1.69	10.13 ± 1.62
Untreated	247.8^a^ ± 15.04	101.4^a^ ± 4.0	28.62^a^ ± 2.13	23.20^a^ ± 2.63
Chrysin	182.9^ab^ ± 34.31	87.25^ab^ ± 3.99	33.50^b^ ± 2.20	17.18^ab^ ± 6.37
Swimming	117.3^bc^ ± 22.66	71.0^bc^ ± 5.40	34.50^b^ ± 3.63	13.05^b^ ± 4.53
Chrysin &Swimming	111.9^bc^ ± 12.23	69.50^bc^ ± 4.44	38.75^bcd^ ± 2.66	8.38^bc^ ± 3.49

HDL-C: High-density lipoprotein (mg/dl).

LDL-C: Low-density lipoprotein (mg/dl).

Data were expressed as Mean ± SD, n = 8 *P* < 0.05 considered significant.

Comparison between different studied groups is carried out using Post Hoc Test (Tukey) for ANOVA test.

^a^Significantly different from Control rats .

^b^Significantly different from Untreated obese rats.

^c^Significantly different from Chrysin treated rats.

^d^Significantly different from Swimming treated rats.

### Liver and kidney function tests

The untreated obese rats showed significantly higher ALT and AST activities compared with healthy control rats. All treated groups showed a reduction in the level of AST activity and a significant reduction in ALT activity compared with the control rats (*P* < 0.05). The best effects were observed in the obese rats treated with chrysin combined with swimming, as shown in Table 6.

**Table 6:** The changes of parameters of liver and kidney function tests parameters in the different studied groups.

Groups	ALT (U/L)	AST (U/L)	Urea (mg/dl)	Creatinine (mg/dl)
Control	47.25 ± 4.46	204.9 ± 9.48	28.25 ± 3.77	0.59 ± 0.05
Untreated	72.50^a^ ± 7.13	271.0^a^ ± 41.93	41.25^a^ ± 2.12	0.69^a^ ± 0.04
Chrysin	64.0^a^ ± 7.23	240.0 ± 27.56	28.63^b^ ± 2.50	0.64 ± 0.02
Swimming	65.13^a^ ± 11.18	241.4 ± 44.2	28.63^b^ ± 2.67	0.67^a^ ± 0.08
Chrysin &Swimming	63.38^a^ ± 8.47	238.4 ± 5.15	26.0^b^ ± 1.60	0.67^a^ ± 0.04

ALT: Alanine Transaminase (U/L).

AST: Aspartate Transaminase (U/L).

Data were expressed as Mean ± SD, n = 8 *P* < 0.05 considered significant.

Comparison between different studied groups is carried out using Post Hoc Test (Tukey) for ANOVA test.

^a^Significantly different from Control rats.

^b^Significantly different from Untreated obese rats.

Untreated obese rats had a significant increase in urea and creatinine levels compared with healthy control rats. All treated groups showed a significant reduction in urea level compared with the untreated group, while all treated groups showed a mild but not significant reduction in creatinine level compared with the untreated group (*P* < 0.05). The best effects were observed in the obese rats treated with chrysin combined with swimming, as shown in Table 6.

### Serum of MDA and TNF-α parameters

The levels of MDA and TNF-α were significantly higher in the untreated obese rats compared with the healthy control group. The obese rats treated with chrysin showed significantly lower MDA and TNF-α levels compared with the untreated group. Also, the obese rats treated with swimming showed a significant reduction in MDA and TNF-α levels compared with the untreated group (*P* < 0.05). The rats treated with combination of swimming and chrysin showed the best results, as it had a significant reduction in MDA and TNF-α levels compared with both chrysin-treated and untreated groups, as shown in Table 7.

**Table 7:** The changes of MDA and TNF-α parameters in the different studied groups.

Groups	MDA (nmole/ml)	TNF-α (pg/ml)
Control	3.24 ± 0.40	25.0 ± 4.44
Untreated	6.33^a^ ± 0.72	63.50^a^ ± 8.70
Chrysin	4.34^ab^ ± 0.72	48.75^ab^ ± 3.85
Swimming	3.38^bc^ ± 0.50	40.50^abc^ ± 5.50
Chrysin & swimming	2.69^bc^ ± 0.32	32.87^bc^ ± 3.31

MDA: Malondialdehyde (nmole/ml).

TNF-α: Tumor necrosis factor alpha (pg/ml).

Data were expressed as Mean ± SD, n = 8 *P* < 0.05 considered significant.

Comparison between different studied groups is carried out using Post Hoc Test (Tukey) for ANOVA test.

^a^Significantly different from Control rats .

^b^Significantly different from Untreated obese rats.

^c^Significantly different from Chrysin treated rats.

### Expression of PPAR-γ in white adipose tissue and skeletal muscles

**Fig. 2:**
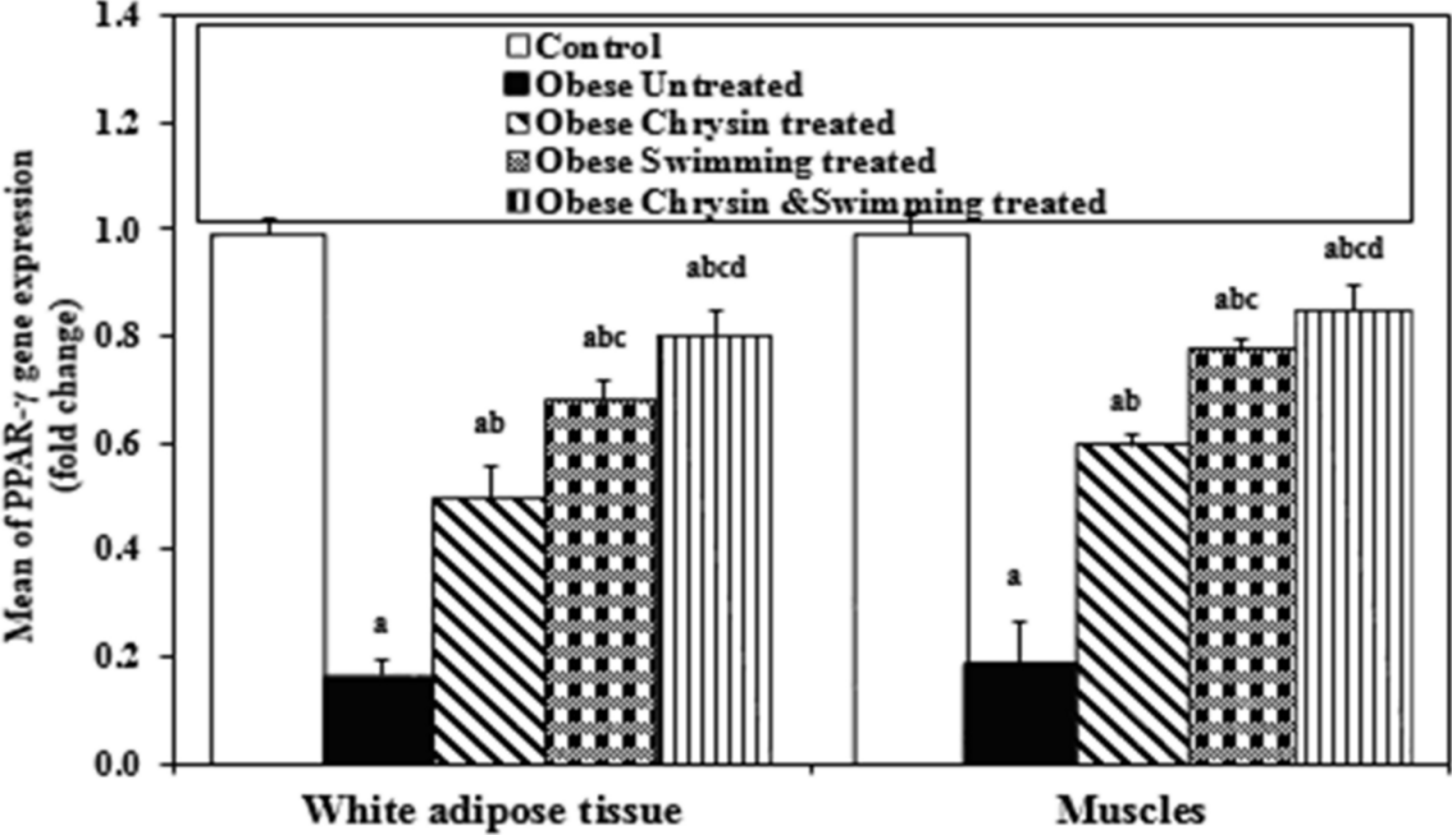
Expression of PPAR-γ in WAT and skeletal muscles (fold change) in the different studied groups. PPAR-γ: Peroxisome proliferator–activated receptor gamma (fold change). Data were expressed as Mean ± SD, n = 8 *P* < 0.05 considered significant. Comparison between different studied groups is carried out using Post Hoc Test (Tukey) for ANOVA test. a: Significantly different from Control rats. b: Significantly different from Untreated obese rats. c: Significantly different from Chrysin treated rats. d: Significantly different from Swimming treated rats.

The results of obese untreated rats showed significant downregulation of PPAR-γ expression as compared with control rats in WAT and skeletal muscles. All treated rats showed a significant upregulation in PPAR-γ expression as compared with untreated obese rats in both tissues. Obese rats treated with swimming and the combined treatment of chrysin and swimming showed a significant upregulation of PPAR-γ expression as compared with chrysin-treated rats in WAT and skeletal muscles. Only the obese rats treated with the combined treatment of swimming and chrysin showed significant upregulation of PPAR-γ expression compared with swimming treated obese rats in WAT and skeletal muscles. Obese rats treated with combined treatment of swimming and chrysin have significant upregulation of PPAR-γ expression especially in muscle tissue but still lower than control (*P* < 0.05), as shown in Fig. 2.

### Expression of Mir27a in white adipose tissue and skeletal muscles

**Fig. 3:**
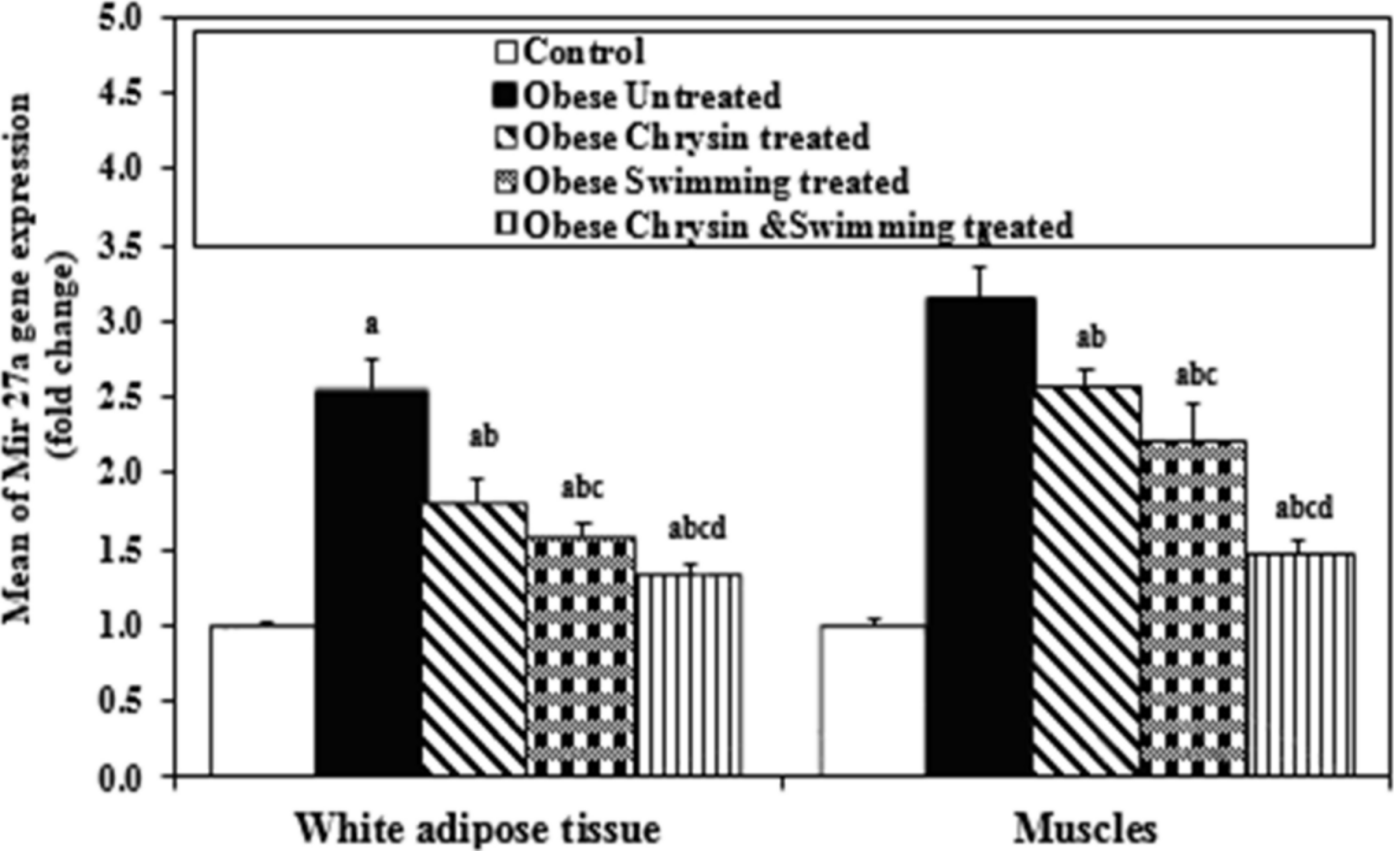
Expression of Mir27a in WAT and skeletal muscles (fold change) in the different studied groups. Data were expressed as Mean ± SD, n = 8 *P* < 0.05 considered significant. Comparison between different studied groups is carried out using Post Hoc Test (Tukey) for ANOVA test. a: Significantly different from Control rats. b: Significantly different from Untreated obese rats. c: Significantly different from Chrysin treated rats. d: Significantly different from Swimming treated rats.

The results of the untreated obese rats showed significant upregulation of Mir27a expression as compared with control rats in both tissues. All treated rats showed a significant downregulation in Mir27a expression as compared with untreated obese rats in WAT and skeletal muscles. Obese rats treated with swimming and the combined group treated with chrysin and swimming showed a significant downregulation of Mir27a expression as compared with chrysin-treated rats in WAT and skeletal muscles. Only the obese rats treated with the combined treatment of swimming and chrysin showed significant downregulation of Mir27a expression compared with swimming-treated obese rats in WAT and skeletal muscles (*P* < 0.05), as shown in Fig. 3.

### Correlation studies

**Fig. 4:**
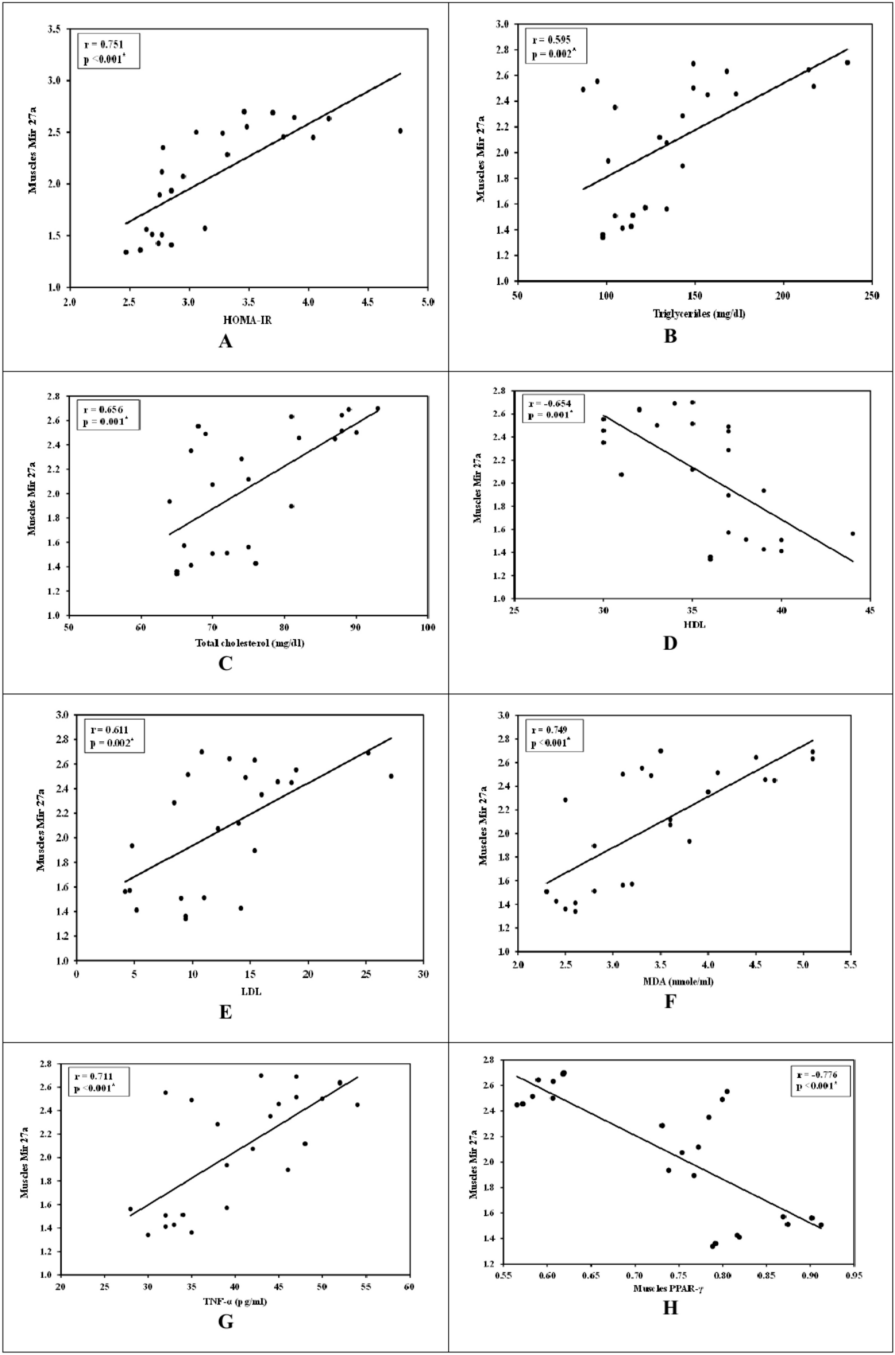
Skeletal muscles correlation studies.

In skeletal muscle tissues:

- Mir27a expression was positively correlated with HOMA-IR (r = 0.751, *P* < 0.001) (Fig. 4A), TG level (r = 0.595, *P* = 0.002),) (Fig. 4B), TC level (r = 0.656, *P* = 0.001) (Fig. 4C), LDL level (r = 0.611, *P* = 0.002) (Fig. 4D), MDA (r = 0.749, *P* < 0.001) (Fig. 4E), TNF-α (r = 0.711, *P* < 0.001) (Fig. 4F) and negatively correlated with HDL level (r = − 0.654, *P* = 0.001) (Fig. 4G) and PPAR-γ (r = − 0.776, *P* < 0.001) (Fig. 4H).

**Fig. 5:**
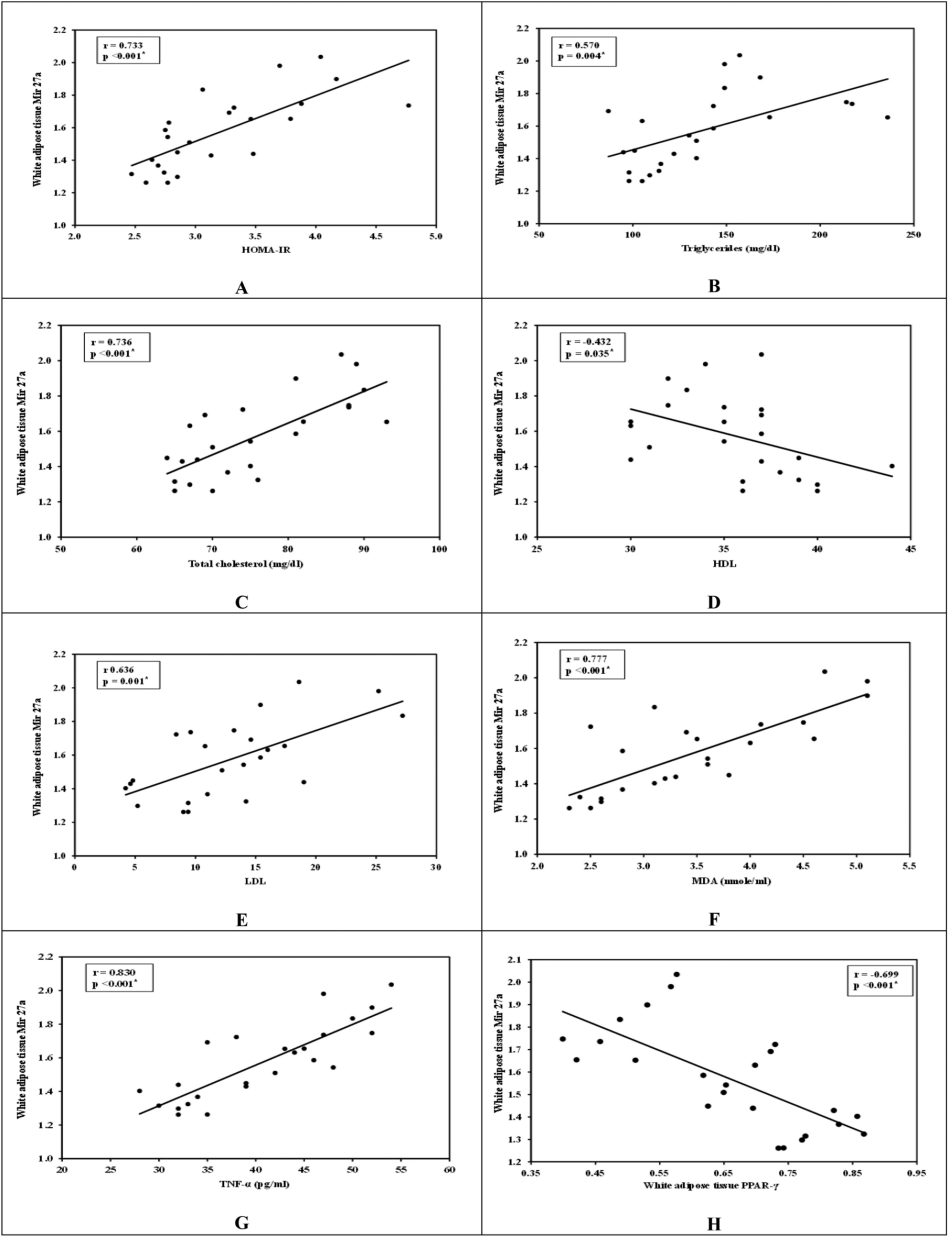
White adipose tissues correlation studies.

In white adipose tissues:

- Mir27a expression was positively correlated with HOMA-IR (r = 0.733, *P* < 0.001) (Fig. 5A), TG level (r = 0.570, *P* = 0.004) (Fig. 5B), TC level (r = 0.736, *P* < 0.001) (Fig. 5C), LDL level (r = 0.636, *P* = 0.001) (Fig. 5D), MDA (r = 0.777, *P* < 0.001) (Fig. 5E) and TNF-α (r = 0.830, *P* < 0.001) (Fig. 5F) and negatively correlated with HDL level (r = − 0.432, *P* = 0.035) (Fig. 5G) and PPAR-γ (r = − 0.699, *P* < 0.001) (Fig. 5H).

## Discussion

The present study showed the potential anti-obesity properties of chrysin compared with physical activity (swimming). This effect occurred through amelioration of carbohydrates and lipid metabolism and correction of the expression of PPAR-γ and miR-27a in both WAT and skeletal muscles in rats with HFD-induced obesity. The HFD-obese rats in the present study showed the main characteristics of obesity, including weight gain, dyslipidaemia, and insulin resistance. These derangements are associated with marked molecular changes, including downregulation of the PPAR-γ gene and upregulation of the expression of miR-27a in both WAT and skeletal muscles. The obese rats have final weights about 60% heavier than the control rats and weight gains about 3.5-fold the control rats during the experimental period. Also, these rats showed insulin resistance that is indicated by elevated HOMA-IR, besides elevated serum liver enzyme activities (AST and ALT) and significantly higher urea and creatinine levels. The present study has shown increased triglycerides, total cholesterol, and LDL levels in the blood, together with a significantly reduced level of HDL cholesterol in untreated obese rats compared to control rats. Kim et al. reported that metabolic abnormalities in glucose metabolism were linked to dyslipidemia. The development of obesity and prediabetes is defined by a vicious cycle that stimulates insulin secretion, insulin resistance and dyslipidemia^24^.

In the present study, the obese group developed a significant elevation in serum TNF-α level compared with the control group. TNF-α, which is known as a proinflammatory cytokine, performs important roles in the pathogenesis of obesity. It was suggested that the concentration of TNF-α in serum increases proportionally to its expression in the adipose tissue. The elevated TNF-α that was associated with insulin resistance has been demonstrated in diverse animal obesity models; it was reported that TNF-α was found to promote insulin resistance^25^.

In the present study, there is an elevation of serum MDA level in a model of obesity. MDA is a byproduct of polyunsaturated fatty acid peroxidation in the cell membrane. A higher blood MDA level is a sign of increased lipid peroxidation and oxidative stress in diabetes and obesity^26^. Accumulation of undesirable or damaged cellular components might enhance the generation of reactive oxygen species or DNA damage, which can ultimately cause cell death^27^.

Collectively, the obese rats in the present study showed many features of obesity, including weight gain, dyslipidaemia, and insulin resistance. An increase in MDA and TNF-α serum levels was observed, which indicated oxidative stress and inflammation. These imbalances were associated with marked changes in both WAT and skeletal muscles, including a significant downregulation of the expression of PPAR-γ and a significant upregulation of the expression of miR-27a genes.

Peroxisome proliferator-activated receptor gamma is a nuclear receptor that controls the transcription of numerous genes linked to diabetes and obesity. To boost the adipose tissue’s fat reserves, it promotes adipocyte development and proliferation. Moreover, it stimulates the activation of lipoprotein lipase (LPL) in adipose tissue, which reduces the quantity of free fatty acids (FFAs) in plasma lipoproteins by engulfing these lipids and storing them as triacylglycerol in adipose tissue. As a result, FFAs decline while the insulin receptor’s sensitivity rises^28^.

Humans with dominant-negative mutations in a single allele of PPAR-γ have partial lipodystrophy and insulin resistance, which is consistent with its crucial involvement in adipogenesis and insulin sensitization. The role of PPAR-γ in insulin sensitization was confirmed by the finding that thiazolidinedione (TZD), which are known to have strong adipogenic and anti-diabetic actions, are agonists for this nuclear receptor. So, the “gold standard” for treating metabolic disorders remains directly targeting PPAR-γ^29^.

In general, PPAR-γ is a potent modulator of whole-body lipid metabolism and insulin sensitivity, making its upregulation a compensatory mechanism to manage excess lipid intake and storage in obesity. Although pharmacological stimulation of only one of these pathways might be enough to produce a therapeutic benefit, the physiological complementarity of these pathways demonstrates the promise of many (and potentially safe) therapeutic intervention approaches^29^.

MiRNA is used as a biomarker for metabolic disorders and obesity. Human body fat percentage and circulating miR-27a levels are highly positively linked. According to this discovery, human adipose tissue may produce and secrete miR-27a. As was already established, miR-27a controls the polarisation of M1-like macrophages, which can promote inflammation in adipose tissue. Additionally, exosome trafficking allows skeletal muscle cells to take up miR-27a that is generated from adipocytes. Exosomal miR-27a from 3T3-L1 adipocytes treated with palmitate also inhibits PPAR γ, causing insulin resistance in C2C12 skeletal muscle cells^30^.

Also, mir27a directly targets PPAR-gamma, and its downregulation leads to increased PPAR-Gamma activity, contributing to enhanced adipogensis and lipid accumulation. This downregulation is associated with increased adiposity and insulin resistance, as mir27a normally acts to repress PPAR-gamma and is also linked to proapoptotic status in adipose tissue, suggesting a role in adipocyte dysfunction and metabolic disorders^31^ and these results were conformed in the present study by correlation Figs. 4H and 5H.

The current study is designed to test chrysin, a natural flavonoid, promising effects on white adipose tissues and skeletal muscle, specifically in the context of treating obesity in rats. In the present study, the effects on rats receiving chrysin orally with a dose of 100 mg/kg daily for two months were observed in comparison with rats performing physical activity (swimming) for 1 h/day, five days per week for two months, with a constant overload equivalent to 5% of their body weight. In addition, there was a combined group treated with chrysin and physical activity.

According to Yao et al., 2021, who investigated toxicological evaluation of a flavonoid, chrysin: morphological, behavioral, biochemical and histopathological assessments in rats, their results determined the LD_50_ value of chrysin to be 4350 mg/kg, whereas no observed adverse effect level (NOAEL) and lowest observed adverse effect level (LOAEL) of chrysin were found to be 500 and 1000 mg/kg, respectively, for both sexes^32^.

The present study showed that chrysin treatment significantly decreased the final weights and weight gains in the obese rats during the period of the experiment. Also, chrysin treatment ameliorates insulin resistance and dyslipidaemia in obese rats. It has been reported that the precise molecular mechanism through which chrysin affects insulin sensitivity is unknown; however, this study of Farkhondeh et al. described the potential effects of chrysin through improvement of the hepatic, pancreatic, and adipose tissue levels of glucose transporters, activation of the components of the insulin signaling pathway, including inhibition of gluconeogenesis, and amelioration of insulin sensitivity^33^. Furthermore, the current study showed that chrysin treatment significantly suppressed the markedly enhanced TNF-α level in obese rats in compared with control, and this may indicate the anti-inflammatory effect of chrysin in obese rats, as has been previously reported in a study by Oriquat et al.^34^.

In accordance with earlier research, it was demonstrated that chrysin suppressed hyperlipidaemia and hyperglycaemia in aged rats through regulating inflammatory responses. Age-related dyslipidaemia, hyperglycaemia, and obesity are mostly caused by chronic low-grade inflammation in tissues. It has been hypothesised that chrysin interacts with several targets related to insulin resistance and obesity. At first, it has been shown that chrysin decreases the levels of TNF-α in many tissues. Second, by preventing IκBα degradation, chrysin can prevent the activation of the nuclear factor kappa B (NF-κB) signalling pathway. Third, by inhibiting IKK, a multi-subunit IκB kinase that activates NF-κB, chrysin can reduce the expression of inflammatory indicators such as vascular endothelial growth factor and cyclooxygenase-2^33^. Additionally, the present findings about MDA level showed a significant decrease in untreated rats as compared with control, and these results were supported by Anand et al., as chrysin treatment prevented oxidative stress-induced tissue damage and changes in a number of parameters, including MDA and antioxidant parameters^35^.

In the present study, obese rats treated with chrysin have a significant upregulation of PPAR-γ expression and a significant downregulation of mir27a expression in WAT and skeletal muscle as compared to obese untreated rats. These results are in line with another study’s observation, as honey, propolis, and other plant extracts have been found to contain chrysin, a natural flavonoid that is a PPAR-agonist. By activating PPAR-γ, chrysin has been proven to reduce inflammation and the vascular problems brought on by insulin resistance^36^.

In the present study, the physical activity (swimming) showed better effect than chrysin alone. The swimming treated group had a significant reduction on final weight, weight gain,LDL, insulin resistance, MDA and TNFα and significantly increased HDL as compared to control group. The most ameliorative effects were noticed in the obese rats treated with chrysin and trained by swimming which completely normalized fasting blood glucose level and HOMA-IR and significantly decreased final body weights and weight gain compared with the control rats. These results are in agreement with Wang et al. which reported that HDL-C are more sensitive to aerobic exercise than both LDL-C and TG. On the other hand , LDL-C consistently decreased after exercise in rats^37^.

Furthermore, the present results agree with the previous finding as the body weight and TNF-α expression in the visceral adipose tissue were all higher in the high-fat diet groups. TNF-α expression was lower in the high-fat swimming group than it was in the high-fat sedentary groups. The outcomes demonstrated that exercise improved lipid profiles, adiposity, and inflammation related to obesity in rats recommending their usage as a substitute to manage the negative effects of a high-fat diet on humans^14^.

TNF-alpha is a pro-inflammatory cytokine that significantly influences insulin resistance and obesity-related signaling pathways. It activates the IκB Kinase (IKK) complex, leading to the phosphorylation and degradation of IκB proteins. This release of NF-κB initiates transcription of pro-inflammatory genes, impairing insulin signaling and contributing to insulin resistance. TNF-alpha also activates several Mitogen-Activated Protein Kinases (MAPKs) pathways, such as p38 MAPK and JNK, which can reduce insulin sensitivity and promote metabolic dysfunction. TNF-alpha can also induce the expression of Suppressor of Cytokine Signaling (SOCS) proteins, which inhibit insulin signaling pathways. Understanding these pathways is crucial for developing therapeutic strategies to mitigate obesity effects and improve insulin sensitivity^38^.

In the current study, in comparison to untreated rats, obese rats treated with physical activity had a significant upregulation of PPAR-γ expression and a significant downregulation of mir27a expression in WAT and skeletal muscle. This effect is still better than chrysin alone and the best results were noticed in the combined group treated with chrysin and swimming in both tissues.

Exercise has an impact on cellular homeostasis and changes the circulating miRNA signature. These dynamic changes in the circulating miRNA profile occur during both the acute reaction and the long-term adaptation to exercise. MiRNAs may be released into the circulation shortly after acute exercise by a variety of cell types, which likely reflects tissue stress or damage brought on by exercise. Moreover, tissue investigations have clearly shown how different types of exercise affect the miRNA profile in the heart, skeletal muscles, and vasculature^39^.

The regulation of body mass might be influenced by the increased expression of miR-27a. MiR-27a derived from these adipocytes induced insulin resistance in skeletal muscle cells through miR-27a-mediated repression of PPAR-γ and its downstream genes involved in the development of obesity^30^. In a clinical investigation, the examination of circulating levels of miRNAs indicated lower plasma levels of miR-27a in obese people in the acute and chronic response of exercise training^40^. The level of serum exosomal miR-27a in the non-exercise obese group was increased obviously, which was reduced in the exercise obese groups. Exosomal miR-27a might be a crucial node for the process of exercise-induced browning of WAT and improving skeletal muscle insulin sensitivity^41^.

In agreement with the present study, Kim et al. reported that high fat diet-induced obese mice, aerobic exercise training increased mitochondrial biogenesis markers more effectively than chrysin supplementation. Additionally, aerobic exercise has a sort of medication to relieve high fat diet-induced metabolic problem^42^.

Lee et al., suggested that by lowering inflammation in some genes, chrysin and moderate exercise have beneficial effects on metabolic problems in obesity caused by high-fat diets. They found that Chrysin supplementation showed little impact on the expression of the thermogenic genes, though. Hence, it would appear that moderate exercise would be more successful in regulating metabolic dysregulation carried on by^43^.

The discussion suggests that chrysin may play a role in altering metabolic processes that are crucial for fat storage and muscle function, making it a promising compound for obesity management in rats, through its effects on these tissues, chrysin could help modulate factors that contribute to obesity, offering a novel approach to treatment strategies in rats.

## Conclusion

From the results of the present study and the above discussion, in obese rats, the upregulation of PPAR-γ and downregulation of Mir27a are interconnected processes that contribute to the pathophysiology of obesity. PPAR-γ upregulation facilitates lipid storage and adipocyte differentiation, while Mir27a downregulation removes its inhibitory effect on PPAR-γ, exacerbating these processes. Understanding these genes expression changes provides valuable insights into obesity-related metabolic disorders and highlights potential targets for therapeutic intervention in rats. It was demonstrated that chrysin influenced the expression of genes involved in lipid and glucose metabolism. Chrysin treatments significantly ameliorate weight gain, hyperglycemia, insulin resistance, and dyslipidemia in obese rats. Also, it is considered a promising factor as an anti-obesity, an antioxidant and anti-inflammatory agent in rats. At the molecular level, chrysin treatments significantly corrected the disturbed expression of PPAR-γ and Mir27a genes in skeletal muscles and WAT tissues. Physical activity (swimming), however, is more effective than chrysin in reducing obesity. Chrysin is proven to be an effective anti-obesity agent, particularly when combined with physical activity for optimal results.

## Data Availability

All data generated or analysed during this study are included in this published article.

## Abbreviations

ALT: Alanine transaminase
AST: Aspartate transaminase
FBG: Fasting blood sugar
HDL-C: High-density lipoprotein
HFD: High fat diet
HOMA-IR: Homeostatic model assessment for insulin resistance
IR: Insulin resistance
LDL-C: Low-density lipoprotein
MDA: Malondialdehyde
MiRNA: Micro ribonucleic acid
mRNA: Messenger RNA
PCR: Polymerase chain reaction
PPAR-γ: Peroxisome proliferator-activated receptor gamma
RNA: Ribonucleic acid
TC: Total cholesterol
TG: Triglycerides
TNF-α: Tumor necrosis factor alpha
WAT: White adipose tissue
WHO: The World Health Organization
